# A Practical Treatise on Nervous Exhaustion

**Published:** 1894-11

**Authors:** 


A Practical Treatise on Nervous Exhauston (Neurasthenia), its Symptoms, Nature, Sequences, and Treatment. By George M. Beard, A. M., M. D., edited with notes and additions by A. D. Rockwell, A. M., M. D. Third edition enlarged. New York: E. B. Treat, 5 Cooper Union. 1894. Price, $2.75.

Every well-informed physician knows this famous production of the late Dr. George M. Beard.

It has now gone through its third edition, showing how well it has maintained its place in the professional regard.

The editor, Dr. Rockwell, was long associated with Dr. Beard, and years ago they jointly wrote a book on electricity in medicine, the best work on the subject that had appeared at that time. This is mentioned merely to show those who may not be acquained with the work now in review that it is not likely to disappoint their expectation in giving clear mental pictures of this very common disease, neurasthenia.

The writer of this notice well remembers the first edition of neurasthenia and the enlightenment obtained by a perusal of its pages.

It is deemed advisable to quote here an extract from Dr. Beard's preface:

"In spite of its frequency and importance, neurasthenia, although long recognized, in a vague way among the people and the profession under such terms as 'general debility,' 'nervous prostration,' 'nervous debility,' 'nervous asthenia,' 'spinal weakness,' and, more accurately, by some of its special symptoms and accompaniments, as 'spinal irritation,' 'nervous dyspepsia,' 'oxaluria,' cerebral and spinal anæmia, and hyperæmia, is even now but just beginning to find recognition in the literature of nervous diseases. It is at once the most frequent, most interesting, and most neglected nervous disease of modern times."

The editor, Dr. Rockwell, in *his* preface says, that it "affords to the profession a convenient refuge when perplexed at the recital of a multitude of symptoms seemingly without logical connection or adequate cause. The diagnosis of neurasthenia, moreover, is often as satisfactory to the patient as it is easy to the physician, and by no means helps to reduce the number who have been duly certified as neurasthenic and who ever after, with an air too conscious to be concealed, allude to themselves as the victims of nervous exhaustion. The doctrine to be taught and strongly enforced is that many of these patients are not neurasthenic, and under hardly any conceivable circumstances could they become neurasthenic."

With these two quotations the reader is left to judge for himself the value of the book in his daily practice.

It may be added, for the information of those who may be specially interested, that this book has been reviewed in The Homœopathic Physician for March, 1889, page 167, and more elaborately in September, 1891, page 374.

